# Autogenous cerebral processes: an invitation to look at the brain from inside out

**DOI:** 10.3389/fncir.2023.1253609

**Published:** 2023-10-19

**Authors:** Pedro E. Maldonado, Miguel Concha-Miranda, Miriam Schwalm

**Affiliations:** ^1^Departamento de Neurociencia, Facultad de Medicina, Universidad de Chile, Santiago, Chile; ^2^Biomedical Neuroscience Institute (BNI), Faculty of Medicine, University of Chile, Santiago, Chile; ^3^National Center for Artificial Intelligence (CENIA), Santiago, Chile; ^4^Bernstein Center for Computational Neuroscience Berlin, Humboldt-Universität zu Berlin, Berlin, Germany; ^5^Department of Biological Engineering, Massachusetts Institute of Technology, Cambridge, MA, United States

**Keywords:** intrinsic brain processes, ongoing activity, closed-loop systems, brain dynamics, autogenous

## Abstract

While external stimulation can reliably trigger neuronal activity, cerebral processes can operate independently from the environment. In this study, we conceptualize *autogenous cerebral processes (ACPs)* as intrinsic operations of the brain that exist on multiple scales and can influence or shape stimulus responses, behavior, homeostasis, and the physiological state of an organism. We further propose that the field should consider exploring to what extent perception, arousal, behavior, or movement, as well as other cognitive functions previously investigated mainly regarding their stimulus–response dynamics, are ACP-driven.

## Introduction

Most neuroscience efforts have focused on understanding the sensory and motor processes necessary to maintain homeostasis. The associated findings have been structured around a conceptual framework in which brain activity primarily results from interactions between the organism and its environment. In this traditional view, neuronal responses in the form of particular firing patterns result from an external stimulus triggering the respective sensory system in the brain. This activity sets off a chain of physiological events through several neuronal circuits, resulting in sensory perception or motor action. Brain function has been extensively studied under this empiricist approach (Yuste, 2015), focusing on neuronal activity as a consequence of external stimulation (Raichle, 2010). However, neuronal activity investigated under this paradigm has continuously revealed intriguing features, such as considerable response variability upon repetitive presentations of the same stimulus. Until fairly recently, this variability was generally referred to either as “noise” or “spontaneous activity” (Stevens and Zador, 1998; Faisal et al., 2008). Central to the “noise” interpretation was the notion that neuronal response variability is primarily a consequence of stochastic processes occurring on different observational scales, from the molecular to the network level (Perkel et al., 1967; Calvin and Stevens, 1968). Further investigation revealed that neuronal variability (Softky and Koch, 1993; Yarom and Hounsgaard, 2011; Uddin, 2020) also exists in the absence of external input (Hartmann et al., 2015) and that it displays properties substantially different from noise (Arieli et al., 1996; Sadaghiani et al., 2010; Uddin, 2020). Two examples of intrinsically emerging activity that were observed and distinguished from background noise in neuronal recordings early on are thalamocortical slow wave events (Steriade et al., 1991) and hippocampal sharp wave ripples (Csicsvari et al., 2000), both related to homeostasis (Fultz et al., 2019) and behavior (Michon et al., 2019).

Using different versions of the classical stimulus–response paradigm has indeed revealed many core principles of neuroscience. It has significantly advanced our knowledge of how neurons in the different loci of the brain respond to sensory stimuli, how motor actions are planned and executed, how this activity is organized, and how it contributes to higher cognitive processes such as perception or decision-making. Due to this legacy, the stimulus–response dyad has justifiably been kept as a standard experimental paradigm in today’s neuroscientific toolkit and still largely dominates our current conceptual framework of brain function (see also Marom, 2020 for a different viewpoint on why this might be problematic). However, focusing solely on stimulus–response-based activity strongly contrasts with our daily experience as active agents exploring and interacting with our surroundings (Maturana, 1970; Varela et al., 1991; Ahissar and Assa, 2016; Haggard, 2017). Naturalistic experimental approaches (König and Luksch, 1998) have revisited the investigation of intrinsic brain processes, especially during task-independent activity (Raichle, 2010, 2015), and concepts of “spontaneous” or “ongoing activity” both assume an active and independent role of the brain (Friston, 2010; Clark, 2013). The theoretical frameworks resulting from these approaches consider the importance of intrinsically initiated neuronal processes and have provided insights into their spatiotemporal representation (Yuste et al., 2005; Deco and Kringelbach, 2017). The study of Maturana and Varela in particular contributed significantly to the concept of the brain as a closed-loop system. They emphasized that the brain operates not as an isolated entity but as an inseparable part of a dynamic feedback loop with its environment (Maturana, 1970; Maturana and Varela, 1987). Maturana and Varela’s contributions challenged traditional views of the brain as a passive recipient of sensory input, paving the way for a more holistic understanding of cognition as an active, self-organizing process deeply intertwined with the outside world.

**Figure 1:**
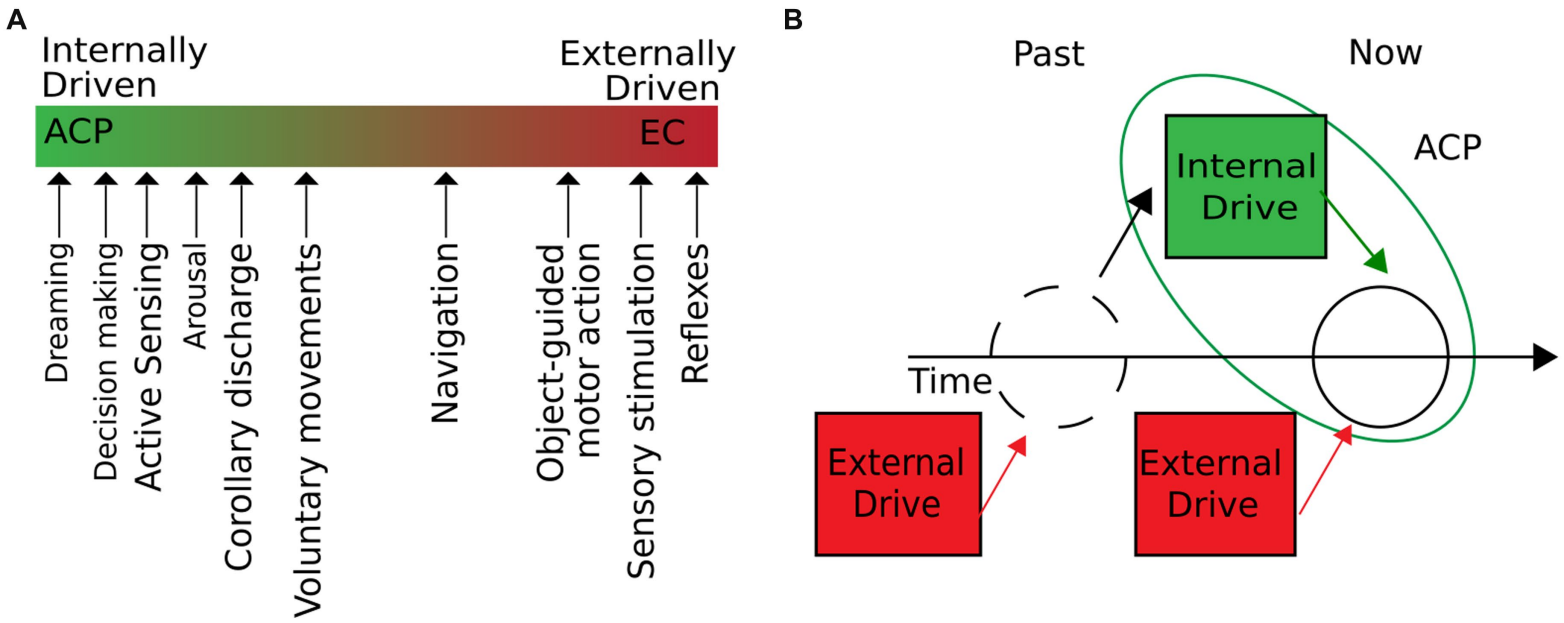
ACPs are intrinsic operations of the brain, initiated independently of the environment. **(A)** We define internal operations as brain processes with little or no influence from external inputs. In natural conditions, the degree to which external and internal drivers influence a brain process can vary. As the figure suggests, different cerebral processes move along the extremes of purely externally or internally driven processes. ACPs would be located on the line’s left (green) side. **(B)** Cerebral processes are initiated independently of the environment when external drivers are not contingent on the development of the process. External drivers may have shaped the system’s state but do not participate in developing the current ACP dynamics.

Building upon these foundations, we propose the concept of autogenous cerebral processes (ACPs) as intrinsic brain operations initiated independently of the environment. ACPs can shape stimulus–response properties and influence a wide range of behavior, homeostasis, and physiological states of an organism, while external input plays only a minor role in their emergence. Our definition of ACPs includes phenomena that under natural conditions occur on a continuum of two extremes: they can either be internally or externally driven and emerge from a combination of internal and external drivers at a given time (Figure 1A). A particular ACP arises when internal drivers are either the only or dominant source (Figure 1B). External drivers comprise inputs outside of the physical boundaries of the brain, such as sensory stimuli or induced changes in homeostasis (e.g., a decrease in oxygen or glucose levels). Internal drivers emerge from within the brain during corollary discharge or top-down control of attention. Since internal drivers can be modulated by external input and vice versa, ACPs are restricted to a particular time window. Consequently, past external influences—although they may have been shaped by internal drivers—are irrelevant to the current ACP. For example, past sensory input can determine the contents of a dream but will not be part of the contingent drivers that initiate the dream. This definition directly implies that different ACPs may be of different durations. Brief internally triggered processes, such as the mentioned corollary discharge, may last only a few milliseconds. Other ACPs, such as the act of dreaming, may have long-lasting inner drivers, extending over several minutes to hours. The central principle to consider is that at a given time, ACPs can occur with little or no sensory–motor interactions. This definition nevertheless implies that ACPs can be *modulated* by peripheral stimulation or environmental coupling as the internal drivers of ACPs can be subjected to external influences.

As such, ACPs underlie perception, arousal, motivated behavior, or movement, while most have been studied primarily regarding their response to external input. However, identifying ACPs is challenging because (i) most experimental paradigms so far are centered on the researchers’ ability to control and trigger a change in neural activity; (ii) different ACPs can be measured by the same proxy (e.g., locomotion or pupil dilation, which in turn have often been assigned to various underlying mechanisms or states, such as arousal, attention, or perception, depending on the scope of the study); and (iii) even when ACPs are successfully captured, their role in explaining behavior has so far likely been underestimated.

To illustrate the concept of ACPs, we here consider the initiation of a motor response. It has been shown that visually guided or object-guided motor action engages brain circuits partially different from the ones associated with internally generated movements (Vaillancourt et al., 2003; Haggard, 2017). It has also been demonstrated that Parkinson’s disease patients show better performance during sensory-guided movements than during internally initiated movements (Majsak et al., 1998). This phenomenon is known as “paradoxical movement” (Glickstein and Stein, 1991) and has been explained as a result of different involvement of basal ganglia circuits in both types of actions (Siegert et al., 2002). A similar distinction has been suggested for externally and internally generated verbal responses, showing different brain activation patterns (Tremblay and Gracco, 2006). These observations suggest that a given behavioral output can result from externally or internally driven mechanisms that recruit different but partially overlapping brain circuits. We propose that a similar organization can be assumed for various phenomena, a perspective highlighting the importance of internally generated processes that our current experimental paradigms may unintentionally conceal.

## Understanding the relevance of ACPs on different observational scales

Concepts that are similar to the definition of ACPs have been explored at the single-cell level (Steriade et al., 2001; Li et al., 2009), but the idea of characterizing internally generated activity has been more traditionally pursued in the context of whole-brain connectivity networks (Raichle, 2010, 2015; Schroeder et al., 2010). However, the absence of a theoretical framework and corresponding terminology hinders our ability to identify and characterize ACPs and their functions. By distinguishing ACPs from classical stimulus-driven processes, our understanding of internal and external drivers of neuronal activity and their interactions might significantly improve. Current methodologies bias neuroscience toward studying behavior and neuronal activity elicited by peripheral stimulation or as a result of long-term steady states. This bias may lead us to overlook changes in brain states with little or no influence from external events, and behavior due to such changes might remain unidentified. We may reveal new insights about their function and origin by applying experimental paradigms that consider internally driven processes. To advance our ability to explain the brain’s essential dynamics, we must introduce a framework incorporating ACPs as distinct and autonomously functioning sources of neuronal dynamics. Previously, some ACPs have been referred to as spontaneous behavior (Stringer et al., 2019), uninstructed movements (Musall et al., 2019), activeness (Gallero-Salas et al., 2021), internal states (Calhoun et al., 2019), or spontaneous activity (Fox et al., 2006). These descriptions refer to observable phenomena that cannot be attributed exclusively to external events. Instead, it is likely possible to explain behavioral outcomes and changes in neural activity with greater precision and accuracy when including these internal constructs (Musall et al., 2019). However, the need to include internal brain dynamics to be able to interpret neuronal signals more precisely contrasts with the lack of a unified theoretical framework describing them. Consequently, we suggest characterizing some of those processes as “inside-out” (Buzsáki, 2019), which may lay the groundwork for building a systematic taxonomy of ACP.

## Examples of presumptive ACPs

The following non-exhaustive list of phenomena illustrates what could be characterized within the proposed ACP framework.

### Perception

To fully understand perception, it is necessary to consider that changes in activity in the sensory organs are frequently a result of self-initiated actions whenever experimental conditions allow for free behavior. This phenomenon has been termed “active sensing,” in contrast to the earlier studied “passive sensing,” the perceptual process resulting from the response to a sudden, unpredicted stimulus (Bajcsy, 1988; König and Luksch, 1998; Bajcsy et al., 2018). For example, in the visual system, active sensing occurs when retinal activity changes due to saccadic movements (Ito et al., 2017). Active perception or active sensing (König and Luksch, 1998)—the original expression was borrowed from the field of machine learning (Bajcsy, 1988)—can be considered an ACP as the associated intrinsic motor activity leads to an active exploration of the environment. For example, the perception of touch requires active movements of the hand (Gibson, 1962; Lederman and Klatzky, 1987) or whisker movements in the case of somatosensory perception in rodents (von Heimendahl et al., 2007; Prescott et al., 2011). This relationship between motor actions (e.g., eye or hand movements) and sensory perception (visual or somatosensory) has been described (Blakemore et al., 2000; Melloni et al., 2009; Wurtz, 2015, 2018) and was shown to be similar for other sensory modalities such as olfaction (Wachowiak, 2011) and audition (Morillon et al., 2015). The principle of motor action modulating visual perception or modulating sensory activity *per se* seems to be a generalized ACP that occurs in humans and other animals, such as rodents, monkeys, fish, and bats (Frens and Van Opstal, 1998; Ulanovsky and Moss, 2008; Stamper et al., 2012; Hofmann et al., 2013; Krieger and Groh, 2015; Pomberger et al., 2020; Umeda et al., 2022). Another well-studied mechanism of a perceptual ACP is corollary discharge. The term was initially proposed by Sperry (1950) and referred to the *“anticipatory adjustment in the visual center specific for each movement concerning its direction and speed.”* Corollary discharge occurs during the initiation of movement, making it a part of active sensing and an ACP, as it can be intrinsically triggered (Poulet and Hedwig, 2002; Schneider et al., 2014). For example, during eye movements, the superior colliculus can modulate activity in visual regions such as the frontal eye field (Sommer and Wurtz, 2006) or in area MT (Berman and Wurtz, 2010). In rats, the secondary motor cortex may modulate secondary visual areas during eye movements (Itokazu et al., 2018). The same regions have been shown to inhibit the auditory cortex during action execution (Schneider et al., 2014), especially for sounds that result from the actions performed (Schneider et al., 2018).

### Arousal

Another putative ACP, arousal, has primarily been studied as a reaction to external stimuli (Ajasse et al., 2018). It has also been examined in the context of internal, higher-order cognitive processes such as visual awareness (Naber et al., 2011), attention (Binda and Murray, 2015; Ajasse et al., 2018), visual memory (Montefusco-Siegmund et al., 2022), emotional perception (Vasquez-Rosati et al., 2017), and working memory (Zokaei et al., 2019), among others (Mathôt, 2018). Low arousal-related inherent signal dynamics are spontaneous and systematic activity during rest periods. These resting-state or default-mode networks were initially used to describe typical functional connectivity patterns between brain regions (Biswal et al., 1995; Raichle, 2011). Although we now understand that spontaneous brain activity fluctuations critically contribute to brain function (Fultz et al., 2019; Uddin, 2020), this activity, often referred to as the ‘global signal,’ is still conventionally treated as noise and typically subjected to artifact removal in resting-state data analysis (Power et al., 2017). Neuromodulation, as a mechanism of regulating arousal, can be considered an ACP as it can intrinsically trigger spontaneous changes or shifts in the functional state (Eggermann et al., 2014; Reimer et al., 2016). More specifically, neuromodulatory transmitters act on target cells in local or large-scale neural networks by affecting synaptic and membrane properties over ion channels, directly influencing conductance, or gaining control of output firing rates (Buzsáki, 2019). The release of neuromodulators such as acetylcholine (ACh), noradrenaline (NE), serotonin, dopamine, and GABA and glutamate upon the external stimulation or animal exploration of their environment has been well studied (Reisine et al., 1982; Sirinathsinghji and Heavens, 1989; Constantinople and Bruno, 2011; Picciotto et al., 2012; Duncan et al., 2014; Eggermann et al., 2014; Hai et al., 2016; Li and Jasanoff, 2020; Breton-Provencher et al., 2021; Mohan et al., 2023). Nevertheless, it has been repeatedly shown that they are also released depending on intrinsic mechanisms, such as “internal clocks” (Kafka et al., 1983; Meck, 1996; Kiehn et al., 2023) and during sleep onset or while waking up (Siegel J, 2004; Siegel J. M, 2004; Lopez-Rodriguez et al., 2007; John et al., 2008; Dash et al., 2009; Berridge et al., 2012; Oh et al., 2019). Notably, these neuromodulation-dependent state changes can be further modulated by the onset of locomotion or other behavioral changes (Poulet and Petersen, 2008; Niell and Stryker, 2010; Bennett et al., 2013; Vinck et al., 2015).

### Motivated behavior

Behavior is a crucial element in maintaining homeostasis and implicates interactions with the environment to seek food, shelter, or interaction with other organisms, regulated by intrinsic reward and metabolic cost. Behavior expresses internal brain dynamics and, as such, is a putative ACP. More specifically, one theoretical perspective proposes that the intrinsic activity of a distributed network, comprising areas such as the ventromedial prefrontal cortex and cingulate, can influence the expression of motivated behavioral drives (Raichle and Gusnard, 2005). Some well-characterized motivated behaviors are thermostasis (Tan and Knight, 2018), food intake (Raynor et al., 2015), and sleep (Vyazovskiy, 2015). One intriguing example of self-rewarded behavior is the act of play. Playful behavior is extensively present across mammals (Iwaniuk et al., 2001), involving either locomotor, sexual, or social behaviors (Burghardt, 2005; Brooks and Burghardt, 2023). Play involves a distributed network comprising cortical and subcortical regions (Siviy and Panksepp, 2011), and an essential characteristic is that it is spontaneous and voluntary (Burghardt, 2005; Brooks and Burghardt, 2023). Rats spontaneously engage in rough-and-tumble play (Panksepp et al., 1984), consistent with its self-rewarding aspect (Calcagnetti and Schechter, 1992). As a strongly self-driven behavior, play is an example of an ACP embodied in a neural network distributed over large areas of the brain. This internally generated process may also arise from state changes in the medial prefrontal cortex and other areas controlling motivated behavior (Raichle and Gusnard, 2005; Reinhold et al., 2019).

### Network dynamics

The idea that the brain’s electrical activity is a continuous and ongoing process was first proposed by Hans Berger at the beginning of the last century (Berger, 1929). Further studies confirmed that even without overt behavior, the brain continues to display coherent activity (Sokoloff, 1977). Arieli et al. (1995) found that the spontaneous firing of single neurons was highly correlated with optical signals and measurements of local field potentials obtained from the cat’s primary visual cortex. Although this activity was spontaneous, it appeared to be highly structured. This idea was reinforced when the same authors reported that ongoing activity, without any given sensory input, dynamically switched cortical states closely corresponding to orientation maps (Kenet et al., 2003). The ACP most studied over the last decade is the already-mentioned default-mode network (Raichle, 2015). This network was defined as a pattern of long-range functional connections between different brain regions that emerged during simple visual fixation or even when participants had their eyes closed. The functional connectivity maps revealed under these conditions differ from the activation observed for attention-demanding, non–self-referential tasks. It has been shown that local neuronal activity (Deco and Kringelbach, 2017) or even the bursting of a single neuron (Li et al., 2009) can influence changes in the global functional brain state through hierarchical processing. Deco and Kringelbach (2017) further argue that intrinsic ignition can describe a brain area’s local capabilities to transmit its activity to other regions in a given functional state. As such, it could occur independently of any sensory process. Thus, intrinsic ignition describes a brain-wide integration of neuronal activity elicited from the propagation of both feed-forward and recurrent activity, making it a definitive example of an ACP.

## Discussion

Essential aspects of brain activity can only be understood by considering intrinsic processes and neuroscience, yet a conceptual framework is needed to comprehensively analyze them. The prevalence of the classical stimulus–response paradigm has led to a reductionist distinction between sensory input and noise, neglecting phenomena that we here summarize under the concept of ACPs. The challenge of unifying disparate evidence within a field of research is ubiquitous in neuroscience. For example, as memory research has grown in complexity, different classifications have helped to bring together seemingly divergent mechanisms under a more comprehensive framework (Johnson, 1983; Squire et al., 1993; Tulving and Craik, 2000). Similar efforts have been made for other cognitive functions by introducing the concepts of top-down and bottom-up-driven perception (Kinchla and Wolfe, 1979; Gregory, 1980; Gilbert and Sigman, 2007; Gilbert and Li, 2013) and, more recently, the distinction between external and internal attention (Chun et al., 2011). Part of what we have learned from these developments is that new conceptual categories can guide scientific efforts toward experimental data that still need a detailed neurobiological explanation. In the context of ACPs, we propose that even though most of the phenomena involving ACPs can be categorized under one of the current theoretical frameworks, i.e., either as a top-down or “internal” process, some of their characteristics are not fully conceptually defined. Despite its enormous flexibility, the bottom-up vs. top-down scheme falls short of incorporating all possible ACPs, mainly because some do not necessarily follow the anatomical architecture proposed for top-down processes. ACPs may not be exclusively embodied in feedback or lateral connections nor do they follow a clear anatomical hierarchy. For example, it is known that the superior colliculus can alter visual processing (Sommer and Wurtz, 2006) or that midbrain areas modulate each other in sensorimotor loops (Mukherjee et al., 2018). In these examples, the characteristic circularity of sensorimotor loops makes it challenging to fit a hierarchy that distinguishes bottom-up from top-down processes. The same difficulty may arise when we study the interaction between higher-order areas, such as the cortex and hippocampus (Siapas et al., 2005), and specific areas in the frontal cortex (Cho et al., 2020). The top-down framework also does not sufficiently emphasize the intrinsic nature of ACPs, since most top-down processes are understood in the context of their bottom-up counterparts. This definition leaves out ACPs such as spontaneous activity, circadian rhythm, dreaming, and, to some extent, voluntary behavior.

### Coda

In this study, we propose to identify a set of brain processes that can occur without the influence of an external drive. We have reviewed some well-known examples, usually studied under stimulus–response paradigms, and suggest that they can also be characterized as autogenous processes (ACPs). Examples of ACPs can be found in the literature under various terms: spontaneous behavior, uninstructed movements, activeness, internal states, active sensing, spontaneous, task-independent, or intrinsic activity. Cognitively complex animals, including humans, behave in a distinctly non-robotic fashion, and several of their high-level behavioral capacities relate to ACPs. Natural and artificial intelligence differences likely originate from ACPs, enriching natural cognition. Thus, complex behaviors such as play and creativity cannot be reduced to stimulus–response contingencies.

**Figure 2:**
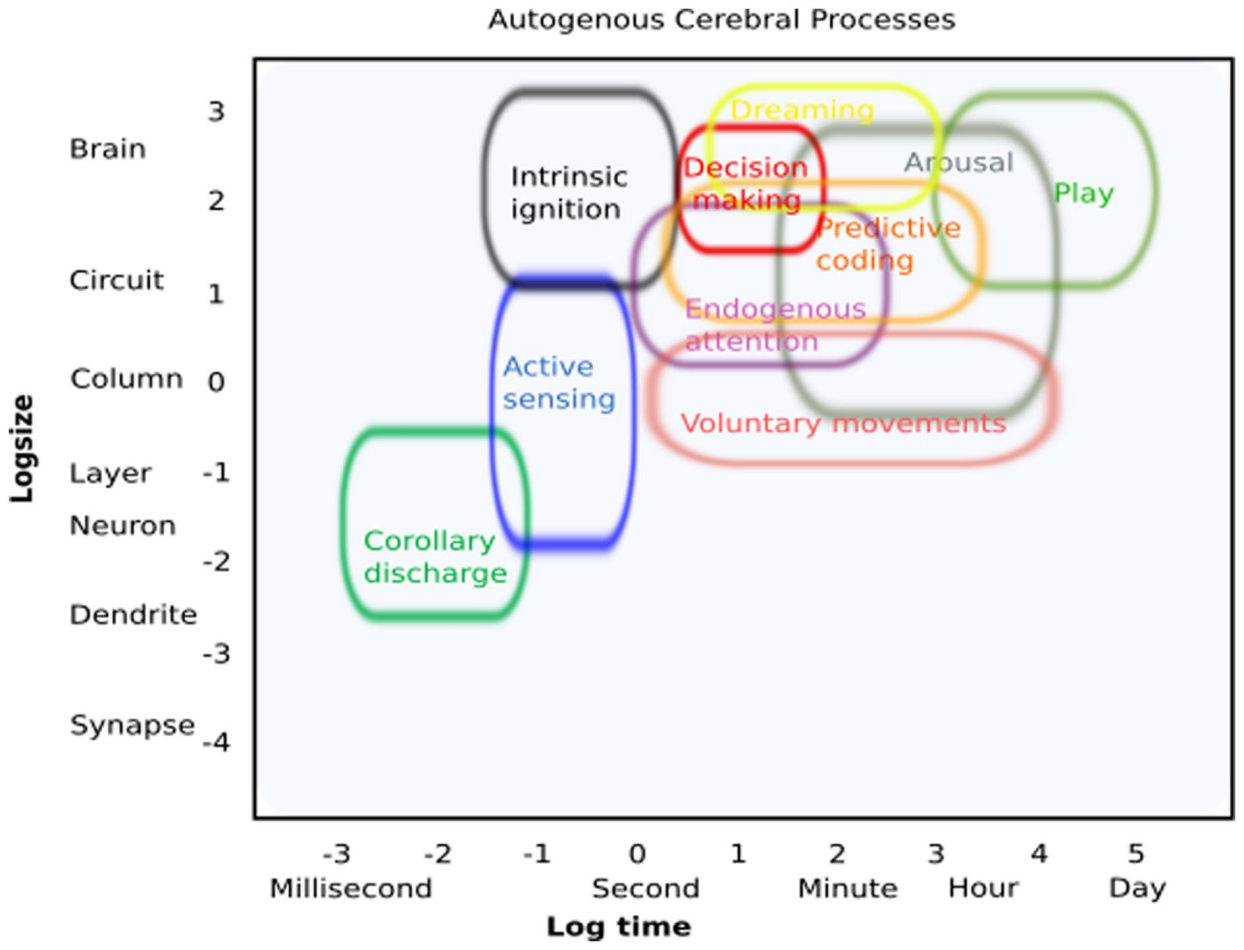
Autogenous cerebral processes: conceptual illustration of how these processes can be identified regarding their putative spatial and temporal resolution.

We borrowed inspiration from Sejnowski (2014), who surveyed current methods of neuroscience and arranged them by spatiotemporal scales, to playfully create an initial conceptual schematic of ACPs and their spatial and temporal resolution (Figure 2). By doing so, we obtained an initial framework to characterize what will eventually reveal the fundamental contributions of ACPs to higher-order cognitive processes and behavior. This may facilitate the study of their commonalities, physiological mechanisms, and potentially distinct functional and structural architectures. As potential new experimental avenues, we suggest the following short-term approaches: (i) changes in experimental design: include free behavior as active sensing or natural behavior (i.e., Markowitz et al., 2023); (ii) including internal processes as explanatory variables, i.e., explaining neural variability or changes in brain dynamics as a result of a process initiated by the organism itself (movement, internally driven changes in a functional state, arousal, or perception); (iii) carefully examine brain dynamics during quiescence states; (iv) explore intrinsic states with the help of deep learning approaches; and (v) identify “spontaneous” but recurring patterns of brain activity that occur in the absence of external stimulation but are highly relevant for brain function (Livne et al., 2022). Following these experimental approaches could reveal the relevance of internal states for explaining cerebral dynamics and behavior beyond the known effects of external manipulation.

## Data availability statement

The original contributions presented in the study are included in the article/supplementary material, further inquiries can be directed to the corresponding author.

## Author contributions

PM: Conceptualization, Writing – original draft, Writing – review & editing. MC-M: Conceptualization, Writing – original draft, Writing – review & editing. MS: Conceptualization, Writing – original draft, Writing – review & editing.

## Funding

This study was funded by the National Center for Artificial Intelligence, CENIA FB210017, Fondecyt N° 1190318 to PM and BNI, Project ACE 210007.

## Conflict of interest

The authors declare that the research was conducted in the absence of any commercial or financial relationships that could be construed as a potential conflict of interest.

The handling editor AN-P declared a shared affiliation with the author PM at the time of review.

## Publisher’s note

All claims expressed in this article are solely those of the authors and do not necessarily represent those of their affiliated organizations, or those of the publisher, the editors and the reviewers. Any product that may be evaluated in this article, or claim that may be made by its manufacturer, is not guaranteed or endorsed by the publisher.
